# Colorectal Cancer Chemotherapy Drug Bevacizumab May Induce Muscle Atrophy Through CDKN1A and TIMP4

**DOI:** 10.3389/fonc.2022.897495

**Published:** 2022-07-01

**Authors:** Qun Xu, Jinyou Li, Yue Wu, Wenjing Zhou, Zherong Xu

**Affiliations:** Department of Geriatrics, The First Affiliated Hospital, Zhejiang University, School of Medicine, Hangzhou, China

**Keywords:** bevacizumab, sarcopenia, TIMP4, CDKN1A, muscles

## Abstract

The muscle in the organism has the function of regulating metabolism. Long-term muscle inactivity or the occurrence of chronic inflammatory diseases are easy to induce muscle atrophy. Bevacizumab is an antiangiogenic drug that prevents the formation of neovascularization by inhibiting the activation of VEGF signaling pathway. It is used in the first-line treatment of many cancers in clinic. Studies have shown that the use of bevacizumab in the treatment of tumors can cause muscle mass loss and may induce muscle atrophy. Based on bioinformatics analysis, this study sought the relationship and influence mechanism between bevacizumab and muscle atrophy. The differences of gene and sample expression between bevacizumab treated group and control group were studied by RNA sequencing. WGCNA is used to find gene modules related to bevacizumab administration and explore biological functions through metascape. Differential analysis was used to analyze the difference of gene expression between the administration group and the control group in different muscle tissues. The key genes timp4 and CDKN1A were obtained through Venn diagram, and then GSEA was used to explore their biological functions in RNA sequencing data and geo chip data. This study studied the role of bevacizumab in muscle through the above methods, preliminarily determined that timp4 and CDKN1A may be related to muscle atrophy, and further explored their functional mechanism in bevacizumab myotoxicity.

## Introduction

Muscle is a major component of lean body mass and plays a vital role in maintaining health. It has been shown that there is a direct or indirect relationship between muscle and strength, energy, mobility, skeletal support, balance, wound healing, immune function, digestive function, and skin health (1).Muscle mass begins to decline from the age of 40 at a rate of 6% per decade and accelerates to a rate of 25–40% per decade above age 70 years. The muscle loss related to age is sarcopenia (2).Sarcopenia in the older adult has been associated with functional impairments, disability, increased risk of falls and fractures, reduced health-related quality of life, and increased risk of death (3). Sarcopenia is considered “primary” when there is no obvious other specific cause, while sarcopenia is considered “secondary” when other causes other than aging are obvious. Sarcopenia can be secondary to systemic diseases, especially those that can trigger inflammation processes, such as malignant tumors or organ failure (4).Sarcopenia in the oncology setting is an area of growing research interest which is associated with many adverse consequences. Its prevalence is high in adult cancer patients, ranging from 11% to 74% in all adults, and even higher in elderly cancer patients (5).

In many clinical studies, skeletal muscle mass (SMM) changes in cancer patients have been extensively investigated through the use of diagnosis, staging, or follow-up computed tomography(CT) scans. Multiple studies have shown that, among cancer patients, the proportion of patients with sarcopenia increased significantly and the skeletal muscle volume and density was lost after treatment with cytoskeletal disruptors (taxanes), nucleotide analogs (gemcitabine) or kinase inhibitors (bevacizumab), or neoadjuvant chemotherapy (6–10).In particular,colorectal cancer patients prescribed bevacizumab appear to lose weight and muscle over a few months even in the absence of cancer progression (11).In colorectal cancer patients with metastatic disease under bevacizumab-based chemotherapy treatment, Adeline et al. assessed changes in muscle mass over an interval of 70 days and indicated 47% patients showed loss of SMM (12). In metastatic colorectal cancer, significant muscle loss occurred in patients with bevacizumab + capecitabine + oxaliplatin-based combination chemotherapy(CAPOX-B) (13, 14). Collectively, the literature supports a direct association between chemotherapy, especially bevacizumab and muscle atrophy.

Bevacizumab (Avastin^®^), a VEGF-A-targeting monoclonal antibody, is the first FDA-approved anti-angiogenic drug in clinical practice. Bevacizumab prevents the interaction of VEGF-A with VEGFR by binding to VEGF-A, inhibiting the activation of VEGF signaling pathway, thereby preventing the formation of new blood vessels (15).Bevacizumab inhibits the growth of human tumor cell lines because of this mechanism. In addition to the above application in patients with colorectal cancer, bevacizumab is also used in the treatment of many other cancers.In the treatment of malignant pleural mesothelioma, bevacizumab combined with pemetrexed and cisplatin can significantly improve the overall survival of patients (16). When combined with paclitaxel, it can improve the curative effect of pleural effusion in non-small cell lung cancer (17).In oral squamous cell carcinoma, bevacizumab was found to downregulate biomarker expression and promote cancer cell apoptosis (18). Given its widespread use in cancer treatment, the cause of bevacizumab-induced sarcopenia needs to be explored urgently.

Mechanisms of action of chemotherapies to induce sarcopenia are various.Chemotherapeutic drugs could induce the expression of pro-inflammatory cytokines, active the myostatin pathway, cause mitochondrial damage and induce oxidative stress.They increase protein degradation by activating ubiquitin-proteasome pathway and autophagy-lysosome pathway, and inhibit protein synthesis by silencing the IGF-1/PI3K/Akt/mTOR anabolic pathway (2). As a kinase inhibitor, bevacizumab inhibits the vascular endothelial growth factor receptor and disrupts many of skeletal muscle pathways, such as PI3K and AKT (11, 19). In this study, we will use bioinformatics approaches to find potential genes associated with muscle atrophy by mouse models with bevacizumab and obtain sequencing information,so as to provide useful help for the clinical use of bevacizumab.

## Methods and Materials

### Data Source

12 mice were tested to obtain a set of sequencing data. Six of the mice were treated with bevacizumab, and the other six were used as the control group. The gastrocnemius muscle and soleus muscle were collected from 6 cases each, and the muscle tissues were collected for RNA-seq sequencing after the same treatment. Finally, MutilQC (https://multiqc.info/) was used to normalize and summarize the data in different samples. Two datasets, GSE38417 and GSE6011, were obtained from the GEO database (https://www.ncbi.nlm.nih.gov/geo/), containing 22 and 37 samples, respectively. Merge GSE38417 and GSE6011 to get a new dataset new_merge.

### Principal Components Analysis

In order to verify the rationality of the data of the 12 mice, PCA was used for cluster analysis to observe the repeatability of the samples. R-packet pheatmap is used to observe the distribution of each sample and gene.

### Weighted Gene Co-Expression Network Analysis

The co-expression of genes in the expression profile was analyzed by R-package WGCNA. The optimal soft threshold is obtained according to the scale-free network(β).According to the similar expression, the genes are divided into different modules, and each color contains different genes. Then analyze the relationship between these gene modules and phenotype, and select the gene module with the most significant correlation with phenotype for subsequent analysis.

### Differential Expression Analysis

R-packet limma packet was used to analyze the differential expression of genes. 12 mouse samples were divided into two independent gene sets GM and SM according to muscle type, and the differences were analyzed respectively to find the genes differentially expressed in the administration group and the control group.| Log2fc | < 1, P < 0.05 was defined as the screening threshold of differential genes.

### Gene Expression

T test was used to analyze the expression of genes between different samples, and R package ggplot2 was used to show the expression of genes. Correlations between genes were analyzed by spearman and plotted by the R package ggstatsplot. In addition, Venn map was used to analyze intersecting genes in different datasets.

### Function Cluster Analysis

Metascape was used to analyze the functions and pathways of key module genes to further understand the relationship between genes and diseases. Single Gene Set Enrichment Analysis (GSEA) was used to analyze the Hallmark pathway and Kyoto Encyclopedia of Genes (KEGG) pathway for the involvement of hub genes in muscle atrophy. The screening conditions for important pathways were |NES|<1, NOM P-val<0.05, FDR q-val<0.25.

## Results

### Verification of Sequencing Data

**Figure 1:**
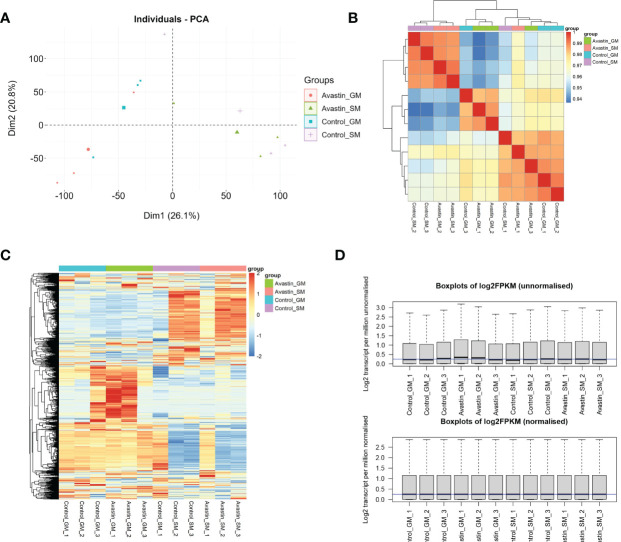
Validation of sequencing data. **(A)** PCA analysis of data distribution in 12 samples; **(B)** correlation analysis of genes in each sample; **(C)** heat map of gene distribution in samples; **(D)** normalization results of samples.

The PCA results showed that the samples in the control group and the administration group could be clearly separated, and the gastrocnemius and soleus muscles could also be clearly separated (**Figure 1A**). Subsequent studies found that both gene correlations and expression distributions were differentiated by sample type (**Figures 1B, C**). Data were normalized for facilitating subsequent studies(**Figure 1D**).

### WGCNA

**Figure 2:**
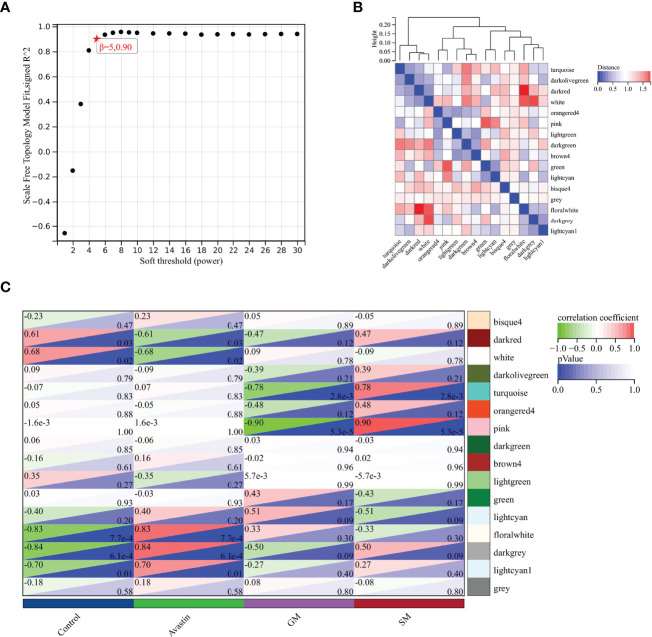
Selection of key gene modules. **(A)** Determination of soft threshold; **(B)** Heat map of module feature vector clustering; **(C)** Heat map of correlation between gene modules and traits.

The optimal soft threshold was determined to be 5 based on standard analysis of scale-free networks (**Figure 2A**). 16 color modules were obtained by WGCNA analysis, and each module contained different genes (**Figure 2B**). By analyzing the correlation between gene modules and traits, it was found that lightcyan1 (cor=0.70, P=0.01), floralwhite (cor=0.83, P=7.7e-4), darkgrey (cor=0.84, P=6.1e-4) had the strongest correlation with bevacizumab administration group (**Figure 2C**). These three gene modules were selected as key modules, containing a total of 280 genes.

### Metascape

**Figure 3:**
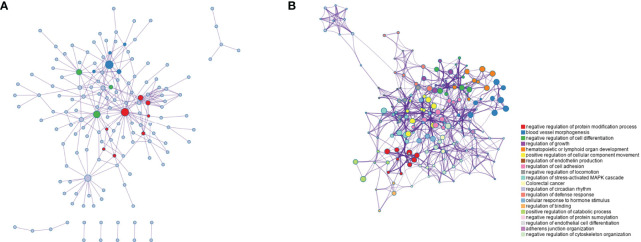
Function clustering analysis of genes in key modules. **(A)** Co-expression network diagram of 280 key genes; **(B)** Rich term network of genes, showing the top 20 functional terms.

As shown in **Figure 3A**,there was the protein-protein interaction network of 280 genes (**Figure 3A**). These genes were subsequently subjected to functional clustering analysis, and most of the genes were enriched in GO functional terms such as negative regulation of protein modification processes, vascular morphogenesis, and negative regulation of cell differentiation (**Figure 3B**). Among the enriched functional terms, 4 functional terms were related to proteolytic pathways. The functional terms related to the proteolytic pathway and the number of genes included in each term are shown in **Table 1**, and there are 39 genes in total.

**Table 1:** Functional terms and number of genes related to MAPK pathway in functional clustering results.

Category	Description	Counts	Hits
GO (BP)	negative regulation of protein modification process	25	Ager|Casp3|Ctnnb1|Cdkn1a|Dmtn|Fkbp8|Hhex|Hmg20b|Irf1|Per2|Inpp5k|Sin3a|Xrcc1|Gadd45g|Sh3bp5|Ceacam1|Tinf2|Ctdsp2|Niban1|Dusp10|Errfi1|Rasd2|Adarb1|Pias3|Kdm4a
GO (BP)	monoubiquitinated protein deubiquitination	3	Taf10|Sf3b5|Asxl1
GO (BP)	positive regulation of protein catabolic process	8	App|Plk2|Tnfsf12|Apc2|Tiparp|Rnf144a|Lpcat1|Trib2
GO (BP)	regulation of protein catabolic process	11	App|Plk2|Tnfsf12|Apc2|Gabarapl2|Tiparp|Rnf144a|Timp4|Lpcat1|Trib2|Ccar2

### Differential Expression Analysis

**Figure 4:**
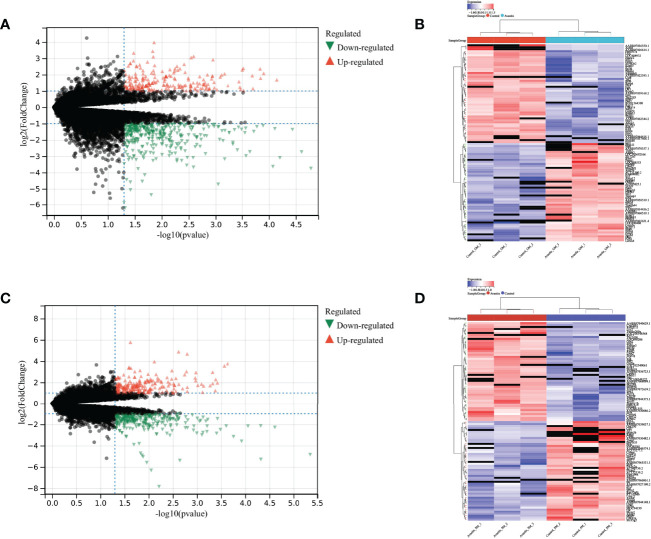
Differential expression analysis results. Differential expression analysis of genes in gastrocnemius muscle, with **(A)** volcano plot showing the differential expression results of genes, **(B)** heat map showing the distribution of up- and down-regulated genes; Differential expression analysis of genes in soleus muscle, with **(C)** volcano plot showing the differential expression results of genes, and **(D)** the heat map showing the distribution of up- and down-regulated genes.

The differential expression analysis between the control group and the administration group in the gastrocnemius muscle showed that 363 genes were down-regulated and 217 genes were up-regulated (**Figure 4A**). At the same time, the difference analysis between the control group and the administration group in the soleus muscle showed that 295 genes were down-regulated and 236 genes were up-regulated (**Figure 4C**). The expressions of up-regulated and down-regulated genes are shown in **Figures 4B, D**, and there are significant differences.

### Gene Expression

**Figure 5:**
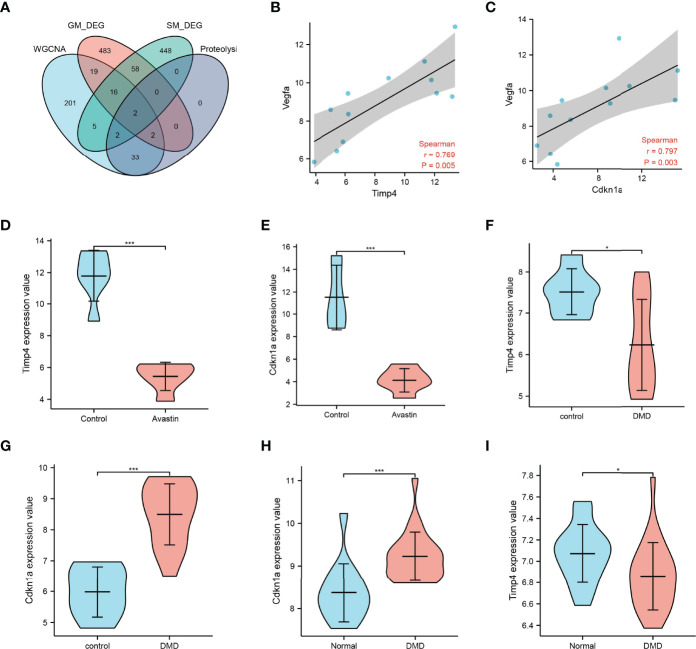
Map3k6 expression. **(A)** Venn map showing the intersection of different datasets; correlation of **(B)** Timp4 and **(C)** Cdkn1a with Vegfa; **(D)** Timp4 and **(E)** Cdkn1a expression in drug-treated and control groups; **(F)** Timp4 and **(G)** Cdkn1a expression in different groups in GSE38417; **(H)** Timp4 and **(I)** Cdkn1a expression in different groups in GSE6011. *p < 0.05,***p < 0.001.

The intersection of key gene modules, GM_DEG, SM_DEG and genes related to the proteolytic pathway in WGCNA, two genes Timp4 and Cdkn1a were obtained (**Figure 5A**). Vegfa is the primary target of bevacizumab. Correlation analysis with Vegfa found that both Timp4 and Cdkn1a were positively correlated with Vegfa (**Figures 5B, C**). The expression comparison showed that Timp4 and Cdkn1a were significantly overexpressed in the control group, but significantly underexpressed in the administration group (**Figures 5D, E**). At the same time, by comparing the expression of Timp4 and Cdkn1a in GSE38417, the results showed the opposite results. Timp4 was underexpressed in the muscle atrophy group, whereas Cdkn1a was overexpressed in the muscle atrophy samples (**Figures 5F–I**).

### Functional Pathways

**Figure 6:**
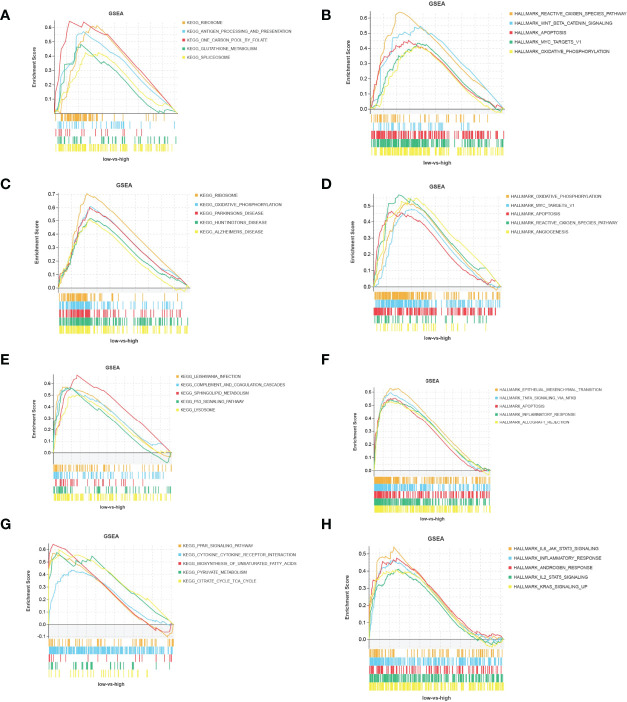
Functional pathways of Timp4 and Cdkn1a. **(A, C)** KEGG pathway and **(B, D)** Hallmark pathway of Timp4 and Cdkn1a in the bevacizumab-administered group; **(E, G)** KEGG pathway and **(F, H)** Hallmark pathway of Timp4 and Cdkn1a in the combined dataset of GSE6011 and GSE38417.

According to the expression of Timp4 and Cdkn1a, the samples were divided into two groups: high expression and low expression, and GSEA was used to analyze the biological signal pathways of gene enrichment. The results showed that Cdkn1a was significantly enriched in KEGG pathways related to ribosomes, antigen processing and presentation, glutathione metabolism,and spliceosome (**Figure 6A**),as well as Hallmark pathways such as reactive oxidation pathways, WNT_β_catenin_signaling,apoptosis, MYC_target_V1,and oxidative phosphorylation(**Figure 6B**). Timp4 was significantly enriched in Hallmark pathways such as oxidative phosphorylation, MYC_target_V1, apoptosis, reactive oxidativepathways,and angiogenesis (**Figure 6C**), as well as KEGG pathways such as ribosomes, oxidative phosphorylation, Parkinson’s disease, Huntington’s disease, Alzheimer’s disease and Hemer’s disease (**Figure 6D**). In the mergeddataset new_merge, Cdkn1a was significantly enriched in Hallmark pathways such as epithelial-mesenchymal transition, TNFA_signaling through NFKB, apoptosis, inflammation and allograft rejection-related pathways (**Figure 6E**), as well as KEGG pathways such as the complement and condensation cascades, sphingolipid metabolism, P53_signaling pathway and lysosome(**Figure 6F**). Timp4 was significantly enriched in Hallmark pathways such as inflammation and inflammatory factor signaling pathways, androgen response and KRAS signaling (**Figure 6G**), and KEGG pathways such as PPAR_signaling pathway, cytokine receptor interaction, pyruvate metabolism, and citrate cycle (**Figure 6H**).

## Discussion

Muscle atrophy is an important manifestation of sarcopenia. Previous studies have shown that pathway changes related to protein ubiquitination and energy production are common features during muscle atrophy (20). In addition, recent studies have also shown that mitochondrial dysfunction (21), inflammatory pathways (22), and drug toxicity are also closely related to the occurrence of muscle atrophy. Bevacizumab was originally used as a first-line treatment for metastatic colorectal cancer and is being continuously investigated for the treatment of non-small cell lung cancer, glioblastoma, renal cell carcinoma, ovarian cancer, cervical cancer and others (15). The use of bevacizumab could increases the risk of several toxicities, including common adverse effects such as bleeding, wound healing complications, gastrointestinal perforation, arterial thromboembolism, hypertension, and proteinuria (23). At the same time,multiple studies have also shown that patients with metastatic colorectal cancer experience loss of skeletal muscle mass during treatment with bevacizumab (24, 25). In addition, lower extremity muscle weakness has also been reported in patients with recurrent glioblastoma (26). In this study, the gastrocnemius muscle (n=6) and soleus muscle (n=6) of mice were used as the research objects, and the blank control group and the bevacizumab administration group were set, and a complete gene expression profile was obtained after normalization. After WGCNA and differential analysis, three sets of key genes were obtained, and the genes related to the proteolytic pathway were intersected. Proteolysis is one of the main mechanisms of muscle atrophy, and two overlapping genes, Timp4 and CDKN1A, were obtained through the venn diagram.

The cyclin-dependent kinase inhibitor 1 (CDKN1A) gene encodes the p21 protein, also known as p21Cip1/WAF1. p21 is considered an important regulator involved in multiple cellular functions, including G1/S cell cycle progression, cell growth, DNA damage, and cell stemness (27). The expression of CDKN1A is related to the progression of various cancers, and it can be initiated by oncogenes, tumor suppressors, inflammatory cytokines, etc., and can inhibit cell proliferation by binding to CDKS, thus inhibiting the growth function of cancer cells (28, 29). In addition to its role in the occurrence, progression and treatment of cancer, CDKN1A has also been proved to be related to muscle atrophy. Wenjing Ma et al. found that CDKN1A was upregulated in denervated skeletal muscle (30). The increase of CDKN1A expression level can also be found in the muscle atrophy model induced by starvation (31).Similarly, the expression of CDKN1A increased in the model of muscle atrophy induced by hindlimb suspension, but decreased after reloading (32). While in other types of skeletal muscle atrophy, such as Duchenne muscular dystrophy (57), amyotrophic lateral sclerosis, aging and critical illness, CDKN1A mRNA is one of the most highly induced skeletal muscle mRNAs (33). As a well-known cell cycle inhibitor, CDKN1A has also been confirmed to be related to cell proliferation and proliferation regulation of muscle cells (34). It has been reported in the literature that Cdkn1a is an important protein related to muscle development, and it is involved in preventing myoblast proliferation, thereby inhibiting myogenesis or regeneration (35). It can affect the repair of skeletal muscle by regulating the expression of CDKN1A (36).However, Daniel K et al. suggested that p21 may promote muscle atrophy through a cell cycle-independent mechanism in skeletal muscle fibers. They speculate that these mechanisms may lead to cellular changes known to promote muscle wasting, including decreased anabolic signaling, increased procatabolic catabolism, decreased protein synthesis, and impaired mitochondrial function (33).In addition, the high induction of p21 during muscle atrophy leads to the decrease of spermine oxidase. Spermine oxidase inhibition mediated by p21 is considered to be a key step in the pathogenesis of skeletal muscle atrophy (37).

CDKN1A (p21) is a downstream target gene of TP53 (p53) (27).The p53/p21CIP pathway is a key pathway that normally responds to persistent DNA damage, and by which cellular senescence is achieved (38).Cell aging inhibits tumorigenesis in the body by preventing the proliferation of potential cancer cells. On the other hand, aging cells will destroy the integrity of local tissues and lead to some pathological changes, such as sarcopenia (39, 40). Cellular senescence has been identified as a mechanism for the development of myopathies associated with muscular dystrophy mouse models (41). Cellular senescence may adversely affect sarcopenia through muscle stem cell dysfunction and a senescence-associated secretory phenotype (38). In addition, other factors that contribute to muscle atrophy are also linked to the p53/p21 pathway.1,25-Dihydroxyvitamin D deficiency induces sarcopenia through the induction of skeletal muscle cell senescence *via* the p53-p21 axis (42). The deficiency of peroxiredoxin 6, an antioxidant enzyme involved in maintaining intracellular redox homeostasis, also leads to the increase of p53-p21 pathway and thereby induces muscle atrophy (43). Doxorubicin (DOX), a chemotherapeutic drug, is an effective cell inhibitor. It also causes myocardial and skeletal muscle atrophy by activating p53-p21 signaling pathway (44). Myostatin also induces the high expression of p21. The excessive induction of p21 will lead to the irreversible senescence in quiescent satellite cell and impaire muscle regeneration (58).

The above studies have shown that high expression of CDKN1A (p21) has adverse effects on muscle. In the present study, CDKN1A showed low expression in the normal muscle group and high expression in the muscle atrophy group; however, the opposite situation occurred when the control group and the bevacizumab-administered group were compared. There are also a few studies that support the benefits of high expression of CDKN1A. The decreased apoptotic susceptibility of myoblasts to ROS is regulated at least in part by enhanced p21 promoter activity and nuclear p21 localization in myotubes (45). Synthetic beta-adrenergic agonists (BA) have broad biomedical and agricultural applications in increasing lean body mass, however BA treatment mediate increased mRNA expression of CDKN1A (46). A study by Diane et al. using clenbuterol to induce muscle hypertrophy also showed that BA treatment resulted in a significant upregulation of Cdkn1a mRNA abundance in skeletal muscle.CDKN1A inhibits cyclin-dependent kinase 2 activity, leading to irreversible cell cycle exit and terminal differentiation of myocytes (47).Satellite cells act as a source of new myonuclei during muscle repair and growth,whereas decreased p21Cip1 in aging skeletal muscle delays the withdrawal of satellite cells from the cell cycle and make them fail to differentiate, resulting in sarcopenia and impaired skeletal muscle regeneration (48). Thus the significant upregulation of Cdkn1a mRNA expression may indicate an increased potential for terminal differentiation and recruitment of myogenic precursor cells to support muscle hypertrophy.

Tissue inhibitor of metalloproteinase 4 (TIMP4) belongs to the family of extracellular matrix metalloproteinase inhibitors and regulates extracellular matrix (EMC) turnover (49). Studies have shown that TIMP4 is involved in cell survival, cell proliferation, inflammation, and epithelial-mesenchymal transition (EMT) signaling networks (50), and knockdown of its expression also indirectly promotes cell invasion and migration (51). The upregulation of TIMP4 gene prevents the metastasis of human cervical cancer cells by inhibiting PI3K/Akt/snail signaling pathway and blocking epithelial-mesenchymal transition (EMT) (52). Extracellular TIMP4 is involved in promoting the activation of the PI3K/AKT/mTOR pathway and promoting tumor metastasis, and is a prognostic and predictive marker for triple-negative breast cancer (53). In addition to this, a new study showed that TIMP4 affects lipid metabolism and smooth muscle cell proliferation (54). However, few studies have been conducted between TIMP4 and skeletal muscle atrophy. Proteomic analysis by Huemer et al. revealed that TIMP4 is a novel marker for the combination of low muscle mass and high fat mass (55). TIMP4 is highly expressed in adipose tissue, and in a study using TIMP4-deficient mice exposed to a high-fat diet, it promoted high-fat-induced obesity, fatty liver, and dyslipidemia. Furthermore, TIMP4-deficient mice are protected from skeletal muscle triglyceride accumulation in the quadriceps (49).The biochemical and structural remodeling of ECM is very important for the normal development of skeletal muscle. Through the study of zebrafish tendon junction (MTJ), Emma et al. found thatTIMP4 plays a regulatory role in this process (56). In this study, compared with normal tissues, TIMP4 showed low expression in both the administration group and muscle atrophy tissues. This suggests that the low expression of TIMP4 may be related to muscle atrophy, and bevacizumab may reduce the expression of timp4.Given that its association with muscle atrophy has not been definitively reported, more research is needed to determine.

## Conclusion

In conclusion, this study confirmed that bevacizumab may have muscle toxicity through bioinformatics analysis. Timp4 and Cdkn1a were identified as key genes, which are of great significance in mouse muscle atrophy. In addition, the biological effects of timp4 and Cdkn1a in the muscle toxicity of bevacizumab were briefly summarized through the analysis of function and pathway, so as to provide useful thinking for the follow-up use of bevacizumab. However, the specific mechanism of bevacizumab on muscle atrophy still needs to be further verified by *in vitro* experiments.

## Data Availability Statement

The original contributions presented in the study are included in the article/supplementary material. Further inquiries can be directed to the corresponding author.

## Author Contributions

All authors listed have made a substantial, direct, and intellectual contribution to the work and approved it for publication.

## Funding

This research is supported by Grant No.2020YFC2005601 from the National Key R&D Program of China and Grant No.81771497 from the National Natural Science Foundation of China

## Conflict of Interest

The authors declare that the research was conducted in the absence of any commercial or financial relationships that could be construed as a potential conflict of interest.

## Publisher’s Note

All claims expressed in this article are solely those of the authors and do not necessarily represent those of their affiliated organizations, or those of the publisher, the editors and the reviewers. Any product that may be evaluated in this article, or claim that may be made by its manufacturer, is not guaranteed or endorsed by the publisher.
